# Disparities in access to health care system as determinant of survival for patients with pancreatic cancer in the State of São Paulo, Brazil

**DOI:** 10.1038/s41598-021-85759-5

**Published:** 2021-03-18

**Authors:** Victor Hugo Fonseca de Jesus, Wilson Luiz da Costa, Laura Carolina Lopez Claro, Felipe José Fernandez Coimbra, Aldo Lourenço Abbade Dettino, Rachel P. Riechelmann, Maria Paula Curado

**Affiliations:** 1 Medical Oncology Department, A.C. Camargo Cancer Center, Rua Prof. Antônio Prudente, 211, São Paulo, SP 01509-010 Brazil; 2 Abdominal Surgery Department, A.C. Camargo Cancer Center, São Paulo, SP Brazil; 3 Pathology Department, A.C. Camargo Cancer Center, São Paulo, SP Brazil; 4 Department of Epidemiology and Statistics-International Research Center (CIPE), A.C. Camargo Cancer Center, São Paulo, SP Brazil; 5 Department of Medicine/Epidemiology and Population Sciences, Baylor College of Medicine, Houston, TX USA

**Keywords:** Cancer epidemiology, Pancreatic cancer

## Abstract

Little is known about the features and outcomes of Brazilian patients with pancreatic cancer. We sought to describe the socio-economic characteristics, patterns of health care access, and survival of patients diagnosed with malignant pancreatic tumors from 2000 to 2014 in São Paulo, Brazil. We included patients with malignant exocrine and non-classified pancreatic tumors according to the International Classifications of Disease (ICD)-O-2 and -O-3, diagnosed from 2000 to 2014, who were registered in the FOSP database. Prognostic factors for overall survival (OS) in the subgroup of patients with ductal or non-specified (adeno)carcinoma were evaluated using Cox proportional hazard model. The study population consists of 6855 patients. Median time from the first visit to diagnosis and treatment were 13 (Interquartile range [IQR] 4–30) and 24 (IQR 8–55) days, respectively. Both intervals were longer for patients treated in the public setting. Median OS was 4.9 months (95% confidence interval [95% CI] 4.7–5.2). Increasing age, male gender, lower educational level, treatment in the public setting, absence of treatment, advanced stage, and treatment from 2000 to 2004 were associated with inferior OS. From 2000–2004 to 2010–2014, no improvement in OS was seen for patients treated in the public setting. Survival of patients with malignant pancreatic tumors remains dismal. Socioeconomical variables, especially health care funding, are major determinants of survival. Further work is necessary to decrease inequalities in access to medical care for patients with pancreatic cancer in Brazil.

## Introduction

In Brazil, pancreatic cancer currently ranks 13th place in incidence and 6th place in mortality^1^, representing 2% of all malignant tumors and being responsible for 4% of all cancer-related deaths^2^. The majority of these cases are concentrated in the Southeast region, particularly in the State of São Paulo^3^. Importantly, it has been shown that the incidence of pancreatic cancer in Brazil has almost doubled in the past two decades^4^, and recent projections foresee further increments in both incidence and mortality in the next 10 years^3^.

Apart from clinical characteristics, such as performance status^5^ and staging at disease onset^6^, many socio-economic variables seem to be related to the prognosis of pancreatic cancer. Studies carried out in developed countries have described worse survival outcomes for patients with lower income^7^, lower education level^8^, rural residency^9^, and non-married marital status^10^. Furthermore, studies have systematically shown that non-Caucasian patients experience inferior survival^11^. Such disparities might be even more evident in Brazil, where health care is provided in three different settings: insurance coverage, public health system, or direct private payment.

Despite abundant information from developed countries, there are no individual-level data on the patterns of survival or health care access of a large group of patients with pancreatic cancer in Brazil. Moreover, we currently do not know which are the most important socio-economic determinants of survival for Brazilian patients with pancreatic cancer. This description is important, as our health system is very complex, with different sources of funding for health care. In this sense, information provided by hospital-based cancer registries can be used to describe the outcomes of this population and their determinants^12^.

Thus, we conducted a retrospective analysis of patients diagnosed with exocrine malignant pancreatic tumors from 2000 to 2014 who were registered in the São Paulo State Health Department database run by Fundação Oncocentro de São Paulo (FOSP). Our primary aim was to describe survival and its determinants. Secondarily, we sought to depict the patterns of health care access of this group of patients.

## Methods

This is retrospective study of patients with the diagnosis of pancreatic exocrine (including non-classified) tumors identified using the network of hospital-based cancer registries run in the State of São Paulo, Brazil. As this is a secondary anonymized data analysis, the need for ethical approval and informed consent term was waived.

### FOSP database

FOSP is an institution administrated by the São Paulo State Health Department and it is responsible to gather, consolidate (exclude duplicate records) and publicize data obtained from health institutions that have hospital-based cancer registries. It is part of the Brazilian network of hospital-based cancer registries that was established in the early 1990s by the Public Health System (SUS—*Sistema Único de Saúde*) and that has adopted an electronical standardized data collection process since 2000. Currently, more than 70 health-care institutions in the State of São Paulo run cancer registries and feed the FOSP database. Most are SUS-affiliated institutions accredited by the State of São Paulo to treat patients with cancer. However, some centers that treat patients almost exclusively in the insurance or private setting participate in the FOSP database as volunteer institutions.

### Patients

We included patients aged 18 years-old and above with malignant (International Classification of Disease[ICD]-O-2 or ICD-O-3 codes/3) pancreatic neoplasms (ICD-10 C25.0–C25.9) diagnosed from 2000 to 2014 with the following ICD-O-2/3 morphologies (codes): acinar carcinomas (8550, 8551), ductal or non-classified (adeno)carcinomas (8010, 8140, 8141, 8190, 8230, 8323, 8440, 8500, 8521), intestinal-type carcinomas (8211, 8260, 8050, 8262, 8450), mucinous carcinomas (8453, 8470, 8471, 8480, 8481, 8490, 8503), adenosquamous/squamous cell carcinomas (8052, 8070, 8073, 8430, 8560), and miscellanea (8452, 8012, 8020, 8021, 8033, 8200, 8251, 8310, 8441, 8510, 8572, 8474, 8575, 8576). Patients with non-classified malignancies (ICD-O-3 8000 and 8001) were included as they most likely represent pancreatic ductal adenocarcinomas (responsible for more than 85% of malignant pancreatic neoplasms) and their inclusion would better translate data of patients with pancreatic cancer at the population level. We included patients with biopsy-proven neoplasms and those in whom diagnosis was based on clinical or laboratory grounds, as defined by the registry centers. We excluded patients with benign or undetermined behavior lesions, as well as those with neuroendocrine, germline, hematological, or mesenchymal neoplasms (ICD-O-2/3 codes 8800 and above). Cases that were misclassified (not pancreatic histologies) were also excluded. A senior pathologist with expertise in gastrointestinal tumors (LCLC) was consulted to settle which morphologies (codes) should be included and how to distribute them into pathologically meaningful subgroups.

### Data extraction, collection, and transformation

Complete databases with individual patient data are available per year of diagnosis from 2000 to 2014. These data can be downloaded free of charge at the FOSP website (http://www.fosp.saude.sp.gov.br/publicacoes/downloadarquivos). Clinical and demographic data included age at diagnosis, gender, source of payment, formal education, extension of disease, and anatomic location. Pathological data comprised pathological confirmation of cancer, pathological type and subtype. To prevent inadequate staging, we standardized the extension of disease throughout the study period based on the TNM (Tumor, Node, Metastasis) description of each one of the three editions used from 2000 to 2014 in the FOSP database (fifth, sixth, and seventh editions)—Supplementary Table 1^13–15^.

### Outcomes

The primary outcome was the overall survival of patients registered in the FOSP database with the diagnosis of pancreatic exocrine (including non-classified) tumors from 2000 to 2014. Overall survival (OS) was defined as the time from the diagnosis to death (from any cause) or last follow-up visit. We also looked for factors associated with survival using Cox proportional hazard models, with emphasis on the source of health care provision (insurance coverage, public health system, or direct private payment).

Secondary outcomes were the patterns of health-care access evaluated by the rates of pathological confirmation of cancer, staging at time of diagnosis, the times from the first visit to specific clinical landmarks (diagnosis and treatment), and the frequency of any (and specific) anti-cancer treatment. Patterns of health care access were also analyzed according to the source of payment in search for differences in health care patterns throughout different economical scenarios. We conducted an exploratory analysis to evaluate the impact of the interactions between time period and source of payment in overall survival.

### Statistical analysis

The distributions of categorical variables were described using relative and absolute frequencies; they were compared among different independent subgroups using Fisher exact test. The distributions of numerical variables were described using median values and interquartile ranges (IQR); they were compared among different independent subgroups using Kruskal–Wallis test. We chose to use non-parametric tests as many numeric variables did not follow the normal distribution. OS was estimated by the Kaplan–Meier method and survival curves were compared using the logrank test. A Cox proportional hazards multivariate model was generated after multiple imputation of missing data with chained equations (further information on “Supplementary Material”) using the following variables: age, gender, formal education, source of payment, extension of disease, treatment, and time period. As sensitivity analyses, we separately repeated the modeling using complete cases only. For this analysis, we checked the assumption of proportionality of hazards using Schoenfeld residuals. We present two-tailed statistical tests and statistical analyses were performed using Stata Version 16 (StataCorp, College Station, Texas—USA).

### Ethical approval and informed consent

Ethical approval and informed consent were waived by the AC Camargo Cancer Center Internal Ethics Review Board as the study used anonymized secondary data. *Methods* All methods were carried out in accordance with relevant guidelines and regulations.

## Results

### Demographical, clinical and pathological characteristics of the study population

Supplementary Fig. 1 portrays the study population flow diagram. Median age was 64 years (IQR: 55–72) and 3623 patients (52.9%) were male—Table 1. The public system was the funding source of health care for 2258 patients (32.9%) and only 697 (10.2%) had a graduate degree. The head of the pancreas was the primary tumor site in 2989 patients (43.6%) and the most common pathological group of tumors was ductal or non-specified carcinoma (N = 5122; 74.7%).

**Table 1:** Demographic, clinical and pathological features of the study population.

	All patient (N = 6855)	Setting		
		Insurance (N = 484)	Public (N = 2258)	Private (N = 199)
**Age (years)**				
Median	64	66	64	66
Interquartile range	55–72	58–73	56–72	58.5–75
Range	19–97	20–94	19–96	29–97
**Gender (%)**				
Male	3623 (52.9)	243 (50.2)	1185 (52.5)	112 (56.3)
Female	3232 (47.1)	241 (49.8)	1073 (47.5)	87 (43.7)
**Payment source (%)**				
Insurance	484 (7.1)	484 (100.0)	0 (0.0)	0 (0.0)
Public	2258 (32.9)	0 (0.0)	2258 (100.0)	0 (0.0)
Private	199 (2.9)	0 (0.0)	0 (0.0)	199 (100.0)
Unknown	3914 (57.1)	0 (0.0)	0 (0.0)	0 (0.0)
**Formal education (%)**				
Illiterate	395 (5.8)	0 (0.0)	136 (6.0)	1 (0.5)
Elementary/Middle School	2938 (42.9)	55 (11.4)	1032 (45.7)	23 (11.6)
High School	767 (11.2)	55 (11.4)	258 (11.4)	42 (21.1)
Graduate degree	697 (10.2)	115 (23.8)	128 (5.7)	106 (53.3)
Unknown	2058 (30.0)	259 (53.5)	704 (31.2)	27 (13.6)
**Anatomic location (%)**				
Pancreatic head (C25.0)	2989 (43.6)	197 (40.7)	1062 (47.0)	82 (41.2)
Pancreatic body (C25.1)	489 (7.1)	67 (13.8)	138 (6.1)	76 (38.2)
Pancreatic tail (C25.2)	330 (4.8)	51 (10.5)	121 (5.4)	25 (12.6)
Pancreatic duct (C25.3)	20 (0.3)	2 (0.4)	9 (0.4)	1 (0.5)
Langerhans' islets (C25.4)	2 (< 0.1)	0 (0.0)	1 (< 0.1)	0 (0.0)
Other specified parts (C25.7)	24 (0.4)	1 (0.2)	6 (0.3)	1 (0.5)
Superposed lesion (C25.8)	198 (2.9)	36 (7.4)	76 (3.4)	5 (2.5)
NOS (C25.9)	2803 (40.9)	130 (26.9)	845 (37.4)	9 (4.5)
**Pathological subtype (%)**				
Epithelial				
Acinar	28 (0.4)	5 (1.0)	4 (0.2)	8 (4.0)
Ductal or non-specified	5122 (74.7)	381 (78.7)	1699 (75.2)	161 (80.9)
Carcinoma				
Intestinal	350 (5.1)	9 (1.9)	87 (3.9)	5 (2.5)
Mucinous	207 (3.0)	28 (5.8)	63 (2.8)	15 (7.5)
Miscellanea	124 (1.8)	33 (6.8)	39 (1.7)	3 (1.5)
Adenosquamous/SCC	43 (0.6)	3 (0.6)	20 (0.9)	3 (1.5)
Non-classified				
Non-classified	981 (14.3)	25 (5.2)	346 (11.8)	4 (0.2)

### Health care access

#### Diagnosis

Table 2 describes the diagnostic features and the time from first visit to diagnosis. Overall, 5933 patients (86.5%) had the diagnosis of pancreatic cancer confirmed by pathology. This happened more often for patients treated in the insurance and private settings (p < 0.001). Excluding those with missing data, 3128 (57.4%) patients had metastatic disease at presentation. Patients treated in the public setting were less likely to be diagnosed with potentially resectable disease (p < 0.001). Median time from the first visit to diagnosis was 13 days (IQR: 4–30). Patients in the public setting experienced significantly longer time from the first visit to diagnosis when compared to those treated in the insurance or private settings (p < 0.001).

**Table 2:** Diagnostic characteristics according to the source of health care payment.

	All patients (N = 6855)	Setting		
		Insurance (N = 484)	Public (N = 2258)	Private (N = 199)
**Pathological diagnosis (%)**^**$**^				
Yes	5933 (86.5)	466 (96.3)	1903 (84.3)	184 (92.5)
No	907 (13.2)	18 (3.7)	353 (15.6)	14 (7.0)
Unknown	15 (0.2)	0 (0.0)	2 (0.1)	1 (0.5)
**Extension of disease (%)**^**&**^				
Potentially resectable	1415 (20.6)	139 (28.7)	396 (17.5)	56 (28.1)
Locally Advanced	907 (13.2)	58 (12.0)	305 (13.5)	16 (0.8)
Metastatic	3128 (45.6)	226 (46.7)	1123 (49.7)	102 (51.3)
Unknown	1405 (20.5)	61 (12.6)	434 (19.2)	25 (12.6)
**Time from first visit to diagnosis (days)**^**#**^				
Median	13.0	10.0	18.0	6.0
Interquartile range	4.0–30.0	3.0–26.0	6.0–38.0	1.0–13.0

^$^p-value for the difference among Insurance, Public, and Private < 0.001 (Fisher exact test).

^&^p-value for the difference among Insurance, Public, and Private < 0.001 (Fisher exact test).

^#^p-value for the difference among Insurance, Public, and Private < 0.001 (Kruskal–Wallis test).

#### Treatment

Table 3 and Supplementary Table 2 describe treatment access and time from first visit to treatment start. Overall, 4813 patients (70.2%) received some sort of anti-cancer treatment. Patients in the insurance setting were more likely to receive some sort of cancer-directed therapy than those in the public setting (p < 0.001). Among those patients who received treatment, the median time from the first visit to treatment start was 24 days (IQR: 8–55). Patients treated in the public setting experienced significantly longer time from the first visit to treatment start when compared to those treated in the insurance or private settings (p < 0.001). Among those who failed to receive anti-cancer treatment, 1428 patients (69.9%) did so because of premature death before treatment start. Moreover, more patients in public setting did not receive anti-cancer therapy as a consequence of early death. Among patients with potentially resectable disease, 1011 (71.4%) were submitted to surgery. Patients treated in the insurance setting were numerically more likely to undergo surgery when compared to those treated in the public or private settings (p = 0.12). For patients with metastatic disease at presentation, 1739 patients (55.6%) received chemotherapy. Patients with metastatic disease treated in the insurance setting were more likely to receive chemotherapy when compared to those in the public and private settings (p < 0.001).

**Table 3:** Treatment characteristics according to the source of health care payment.

All patients	All patients (N = 6855)	Setting		
		Insurance (N = 484)	Public (N = 2258)	Private (N = 199)
**Any treatment (%)**^**$**^				
Yes	4813 (70.2)	419 (86.6)	1469 (65.1)	115 (57.8)
No	2042 (29.8)	65 (13.4)	789 (34.9)	84 (42.2)

Treated patients	All patients (N = 4813)	Insurance (N = 419)	Public (N = 1469)	Private (N = 115)
**Time from first visit to treatment start (days)**^**&**^				
Median	24.0	20.0	36.0	6.0
Interquartile range	8.0–55.0	8.0–35.0	13.0–73.0	1.0–17.5

Non-treated patients	All patients (N = 2042)	Insurance (N = 65)	Public (N = 789)	Private (N = 84)
**Reason for no treatment (%)**				
Refusal	9 (0.4)	1 (1.5)	4 (0.5)	0 (0.0)
Poor clinical status	281 (13.8)	17 (26.2)	201 (25.5)	2 (2.4)
Comorbidities	9 (0.4)	1 (1.5)	3 (0.4)	0 (0.0)
Treatment abandonment	8 (0.4)	2 (3.1)	2 (0.3)	1 (1.2)
Death from cancer	1428 (69.9)	30 (46.2)	487 (61.7)	7 (8.3)
Death from other causes	41 (2.0)	0 (0.0)	16 (2.0)	0 (0.0)
Other	226 (11.1)	14 (21.5)	76 (9.6)	74 (88.1)
Unknown	40 (2.0)	0 (0.0)	0 (0.0)	0 (0.0)

^$^p-value for the difference among Insurance, Public, and Private < 0.001 (Fisher exact test).

^&^p-value for the difference among Insurance, Public, and Private < 0.001 (Kruskal–Wallis test).

### Survival analysis

Median follow-up was 64.7 months (95% CI 61.0–74.1). Median follow-up for patients treated in the private setting was significantly lower than the ones of those treated in the insurance or public settings—Supplementary Table 3. For all 6855 patients, 6,115 overall survival events were registered, with a median overall survival of 4.9 months (95% CI 4.7–5.2). Rates of 1-year, 3-year, and 5-year OS were 26.3, 8.1, and 4.7%, respectively.

Supplementary Table 4 describes unadjusted overall survival estimates according to patients’ and tumors’ characteristics. Patients treated in the public setting experienced worse unadjusted overall survival, regardless of the disease stage—Supplementary Table 5. Patients with acinar and mucinous histologies had numerically improved survival compared to those with ductal or non-specified carcinoma. Conversely, patients with adenosquamous/squamous cell carcinomas had numerically inferior survival compared to those ductal or non-specified carcinoma. Given the low number of patients with some of the pathological subtypes, we did not include this variable in the multivariate analysis. In the Cox proportional hazard model using multiple imputation, advanced age, male sex, treatment before 2010, lower educational status, treatment in the public setting, more advanced disease, and lack of anti-cancer treatment were significantly associated with inferior overall survival—Table 4. Similar results were found in the complete case analysis—Supplementary Table 6.

**Table 4:** Cox proportional hazard regression for overall survival (multiple imputation of missing data; N = 6855).

Variable	Multivariate analysis		
	HR	95% CI	p
**Age group (years)**			
**< **50	1.00		
50–59	1.10	1.01–1.21	0.032
60–69	1.16	1.06–1.27	0.001
≥ 70	1.29	1.18–1.41	< 0.001
**Gender**			
Male	1.00		
Female	0.89	0.84– 0.94	< 0.001
**Period**			
2000–2004	1.00		
2005–2009	1.04	0.96–1.12	0.303
2010–2014	0.90	0.86–1.00	0.046
**Formal education**			
Illiterate	1.00		
Elementary/Middle	1.04	0.92–1.17	0.527
School			
High School	0.92	0.79–1.06	0.249
Graduate degree	0.82	0.72–0.94	0.005
**Source of payment**			
Insurance	1.00		
Public	1.30	1.15–1.47	< 0.001
Private	0.61	0.45–0.83	0.002
**Extension of disease**			
Potentially resectable	1.00		
Locally advanced	1.63	1.49–1.79	< 0.001
Metastatic	2.17	2.01–2.35	< 0.001
**Treatment**			
No	1.00		
Yes	0.38	0.36–0.41	< 0.001

**Figure 1:**
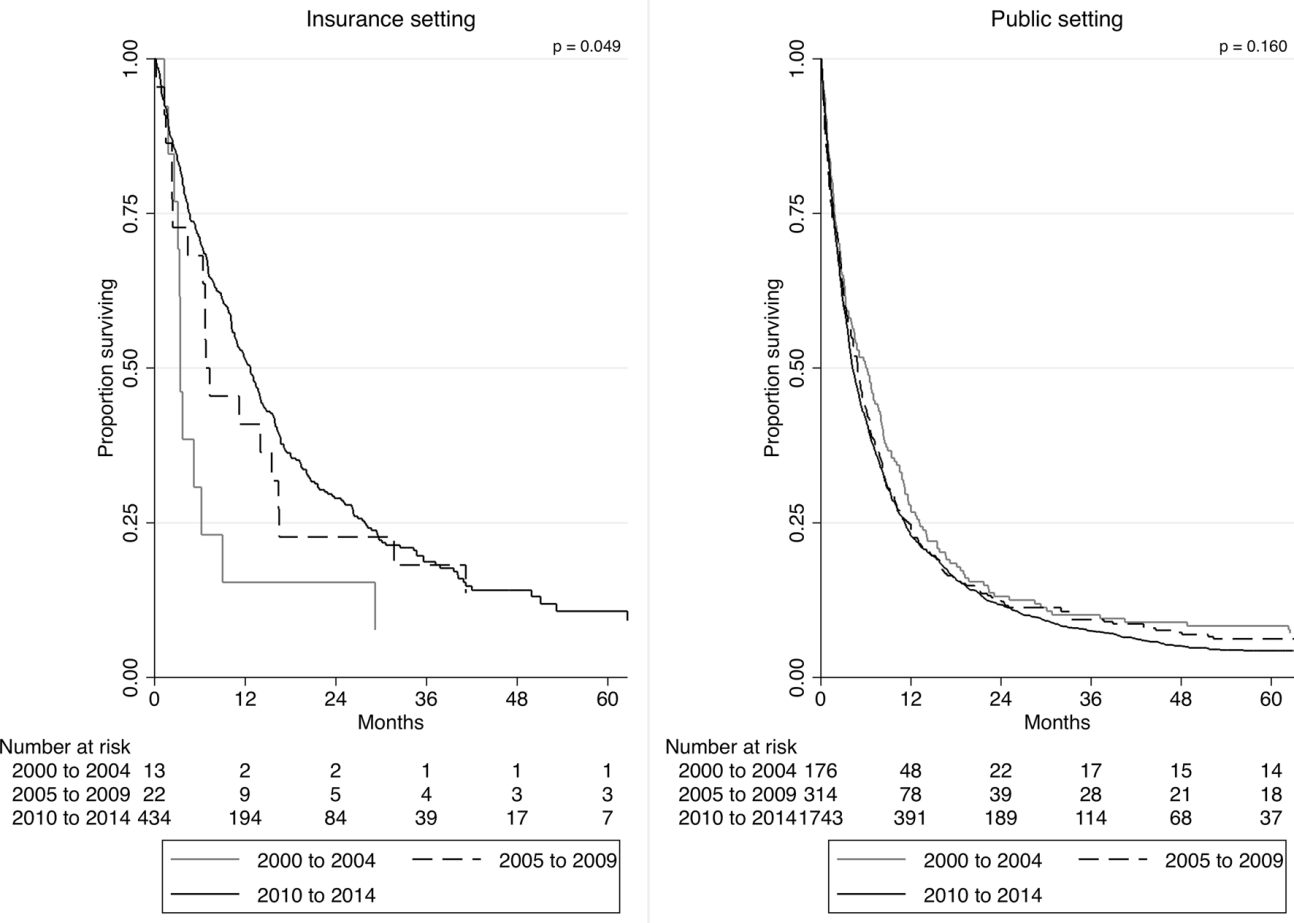
Trends in overall survival in the 2000–2004, 2005–2009, and 2010–2014 periods according to source of payment (insurance or public).

**Figure 2:**
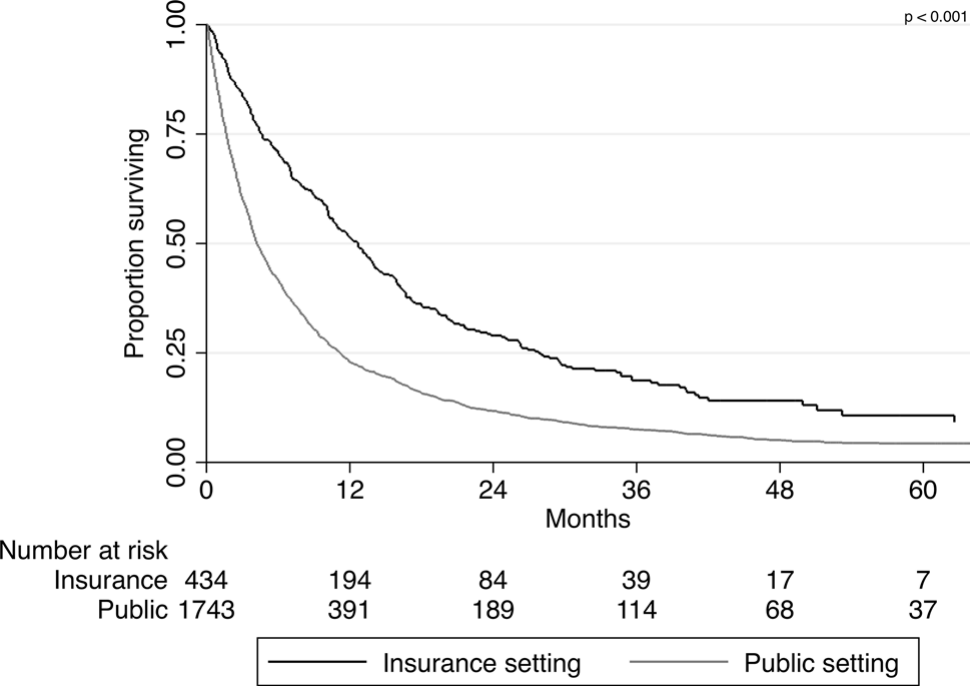
Overall survival in the 2010–2014 period according to source of payment (insurance or public).

We analyzed trends in overall survival from 2000–2004 to 2010–2014 according to the source of payment. Given the low number of patients treated in the private setting before 2010 (N = 4), we restricted the analysis to patients treated in the insurance or the public setting. While there was a significant improvement in overall survival from the 2000–2004 to 2010–2014 for those treated in the insurance setting (p = 0.049), there was no evidence of survival improvement during the study time span for those treated in the public setting (p = 0.160)—Fig. 1. Also, most patients treated in the insurance setting were diagnosed from 2010 to 2014. Analyzing only this period, patients treated in the public health system experienced significantly inferior median overall survival when compared to those treated in the insurance setting (12.7 vs. 4.2 months; p < 0.001)—Fig. 2.

## Discussion

In this study, we describe the outcomes, the determinants of survival, and the patterns of health care access of patients with malignant pancreatic tumors treated in the State of São Paulo from 2000 to 2014. We estimate that these patients represent roughly 20–25% of all cases of pancreatic cancer occurring in this period in the State of São Paulo. These data are important since São Paulo is the most populated State in Brazil, concentrating approximately one fifth of the country’s population.

We observed significant differences in the health care access among patients with pancreatic cancer, mainly due to differences in health care funding. In 2019, 76% of the Brazilian population was uninsured and such patients are almost exclusively treated by the public health system (SUS—*Sistema Único de Saúde*). In our study, patients treated in the public setting were less likely to have a pathologically confirmed diagnosis of pancreatic cancer or to receive any kind of cancer-directed therapy, took longer to be diagnosed or to start treatment, and were diagnosed at more advanced disease stages. Even though the impact of diagnostic and treatment delays in the outcomes of pancreatic cancer patients is debatable^16–20^, the fact that less patients treated in the public setting had their disease diagnosed in the resectable scenario, that these patients were less likely to receive anti-cancer therapy, and that they had inferior survival on adjusted analyses suggest that diagnostic and treatment delays might be harmful.

At first impression, the data on the health care assess in this population seem adequate in light of the results of investigations conducted in developed countries. Such studies have reported lower rates of pathological confirmation of cancer, ranging from 53 to 91%^21^, and median times from referral to treatment start of at least 30 days^16,18^. Also, in developed countries there is evidence that as few as one third of patients with pancreatic cancer are submitted to anti-cancer therapy^22,23^. However, one must consider that much of these data come from population-based cancer registries, and those results should not be directly compared with those from hospital-based cancer registries, as patients in the latter databases are selected in the sense that they have had access to health care, sometimes in regionally renowned institutions. Therefore, we think that the real measures of health care access of patients with pancreatic cancer in the Brazilian general population are inferior to the ones reported herein.

Median overall survival times less than 4 months and actuarial 5-year survival rates less than 10% have been reported in population-based registries in developed countries^21,24,25^. While our study endorses the validity of these data, it also ratifies that the survival of real-world patients with pancreatic cancer is far worse that that reported in randomized trials^26–28^ and that efforts should be concerted to translate potential survival gains seen in clinical trials into improved outcomes for patients with pancreatic cancer at the population level.

In our study, many factors were associated with overall survival. While the differences in survival secondary to more advanced disease and lack of treatment are quite simple to explain, other factors associated with survival deserve mention. In clinical practice the role of age in the prognosis of patients with pancreatic cancer is questionable^29^. However, populational studies have shown that older patients are at greater risk of death, possibly because of the physiological interactions of advanced age and comorbidities with pancreatic cancer^7,30^. Interestingly, epidemiological studies have shown that women have improved survival compared to men. Currently, the reasons for this are unclear. Possibly, hormonal and genetic determinants of disease evolution might differ between genders^7,24,30^. Also, women might be more prone to seek medical advice earlier than men due to social and behavioral reasons^31,32^.

Remarkably, many socio-economic factors were associated with overall survival. We showed that a higher education level was associated with improved outcome. This is in line with previous data demonstrating a clear relationship between level of education and survival in many cancer types, including pancreatic cancer^8^. This might be a consequence of increased awareness to subtle symptoms and prompt search for medical care. Also, patients with higher educational levels are less likely to present comorbidities, which could facilitate adequate treatment of pancreatic cancer^33^. A lower educational status might make communication more complicated^34^, especially in the public setting, where doctors have limited time during the visit to discuss prognosis, treatment plans, and toxicities. In this regard, studies in other tumor sites performed in lower to middle-income countries have shown that interventions targeting the general public and health care workers can improve surrogate health outcomes^35^. Thus, we believe that a great deal of importance must be given to adequately deliver information about cancer to the general population and patients, especially for those with lower education levels, in pursuance of less inequalities in outcomes.

Perhaps more important than the educational level is the source of payment for health care. We were able to show that patients with insurance coverage fared better than those treated in public setting, regardless of the disease stage. There are many possible explanations to this fact. First, there has been no increase in funding for the systemic treatment of individual patients with pancreatic cancer at least since 2008, despite constant rises in the prices of commonly used drugs, such as Gemcitabine^36,37^. As a consequence, more effective chemotherapy regimens such as FOLFIRINOX or Gemcitabine plus Nab-Paclitaxel are seldom used in this setting. Also, longer times to diagnosis and treatment start might have led patients treated in the public system to have a diagnosis in more advanced stages and to have less clinical performance to undergo anti-cancer treatment, thus resulting in inferior survival. Importantly, many patients were treated in institutions with a low mean number of cases of pancreatic cancer per year. From 2000 to 2014, roughly 25% of all patients with pancreatic cancer were treated at UNACONs (High complexity assisting oncology unit), which are institutions qualified to manage the five most common cancers in Brazil, but not pancreatic cancer. These institutions had an average of three cases of pancreatic cancer per year in the same period^38^. While it is possible that the number of patients treated per year in these institutions is underestimated, this average number is far from optimal. It is largely known that, especially in the potentially resectable setting, treatment at high-volume units has been associated with improved survival for patients with pancreatic cancer^39–41^. Therefore, issues related to insufficient health budget and lack of treatment centralization might have contributed to inferior survival for patients treated in the public health system.

Similar to other studies, we have found slight improvements in survival for patients with pancreatic cancer in the past decade^24,25^. Multivariable analyses showed that survival for the 2010–2014 period was longer than that of 2000–2004. However, one interesting finding is that this was not true for patients treated in the public setting. In this group, survival remained essentially unchanged throughout the study period, a finding that probably reflects the lack of technology incorporation secondary to budget restrictions.

Our study presents some limitations. First, some variables have a significant proportion of missing data. However, we addressed this issue by generating a model based on multiple imputation of missing data. Moreover, results of the complete-case analysis showed very similar results, demonstrating the robustness of our findings. Second, the effect of some variables in survival did not follow the proportional hazards assumption in the complete-case analysis. Nonetheless, we believe the conservatism principle may be used in this situation as these variables were considered to be important determinants of survival in our study^42^. Third, patients treated in the private setting had significantly shorter follow-up and higher censoring rates when compared to patients treated in other settings. We speculate that many patients start treatment at private hospitals and then are forced to move to other institutions when they can no longer afford to pay for the continuation of treatment. For that, we believe that caution is recommended when analyzing patterns of treatment access and survival results for this group of patients. Forth, data on the frequency of risk factors for pancreatic cancer, such as smoking and alcohol consumption, along with information on self-reported ethnicity and marital status, were not available in the FOSP database and this hindered a better characterization of the population. Last, we had no data on the specific treatments (e.g.: type of surgery or chemotherapy) used. However, our study also has important virtues. It is, to our knowledge, the largest survival analysis of patients with pancreatic cancer in Brazil. We were able to evaluate survival separately for patients with malignant exocrine tumors according to pathological subtypes. Most importantly, the results of our regression models highlight the weight of different determinants of survival in our population and, along with the data on the patterns of health care access, can aid the elaboration and implementation of effective measures to improve the survival of Brazilian patients with pancreatic cancer.

To conclude, the prognosis of exocrine pancreatic cancer in the State of São Paulo for those patients treated at institutions accredited by the FOSP remains poor. Patients treated for pancreatic cancer in the public setting wait longer to diagnose and to treat pancreatic cancer. They are less likely to undergo anti-cancer treatment and they are at greater risk of early death from pancreatic cancer. From our work, we suggest that increased societal awareness about the disease, treatment centralization, timely access to standard treatment approaches, and improved communication between physicians and patients will likely contribute to improved survival.

## Supplementary information

Supplementary information.

## Data Availability

The datasets generated during and/or analyzed during the current study are available from the corresponding author on reasonable request.

## Abbreviations

FOSP: Fundação Oncocentro de São Paulo
SUS: *Sistema Único de Saúde*
ICD: International Classification of Diseases
UNACON: High complexity assisting oncology unit
OS: Overall survival
IQR: Interquartile range
