# Supplementary material for: Disparities in access to health care system as determinant of survival for patients with pancreatic cancer in the State of São Paulo, Brazil
Source: Sci Rep. 2021 Mar 18;11:6346. doi: 10.1038/s41598-021-85759-5 (PMC7973503; doi:10.1038/s41598-021-85759-5)
Supplement: Supplementary file 1 — Supplementary information. [file 41598_2021_85759_MOESM1_ESM.docx]

**Supplementary information**

**Disparities in access to health care system as determinant of survival for patients with pancreatic cancer in the State of São Paulo, Brazil**

**Authors:** Victor Hugo Fonseca de Jesus, MD, MSc^1^, Wilson Luiz da Costa Jr, MD, PhD, MPH^2,5^, Laura Carolina Lopez Claro, MD^3^, Felipe José Fernandez Coimbra, MD^2^, Aldo Lourenço Abbade Dettino, MD, PhD^1^, Rachel P Riechelmann, MD, PhD^1^, Maria Paula Curado, MD, PhD^4^

Table of content

[Statistics 3](#_Toc47427942)

[Supplementary tables 4](#_Toc47427943)

[Supplementary figures 10](#_Toc47427944)

[References 11](#_Toc47427945)

# **Statistics**

**Multiple imputation**

Variables with missing data were formal education, source of payment, and staging. We used MICE (Multiple Imputation with Chained Equations) to impute data (for the variables Formal education, Source of payment, and Extension of Disease) using the following variables as predictors: age group, gender, period of time, treatment, death (censoring event), and cumulative hazard (using the Nelson-Aalen estimator).^1,2^ We applied augmented regression to avoid perfect predictions. Formal education and extension of disease were predicted based on ordinal logistic regression and Source of payment was predicted based on multinomial logistic regression. We generated 20 imputed models (N = 20). After that, coefficients were pooled using standard techniques.^3^

# **Supplementary tables**

**Supplementary table 1.** Standardization of the extension of disease for pancreatic cancer across different AJCC editions.

| **Abbreviated staging** | **AJCC 5th edition^4^** | | | | **AJCC 6th edition^5^** | | | | **AJCC 7th edition^6^** | | | |
| --- | --- | --- | --- | --- | --- | --- | --- | --- | --- | --- | --- | --- |
|  | 2000-2005 | | | | 2006-2013 | | | | 2014 | | | |
| Potentially resectable | Stage I  Stage II  Stage III | | | | Stage I  Stage II | | | | Stage I  Stage II | | | |
| Locally advanced | Stage IVA | | | | Stage III | | | | Stage III | | | |
| Metastatic | Stage IVB | | | | Stage IV | | | | Stage IV | | | |
| **TNM** | | | | | | | | | | | | |
| **TNM - T** |  | | | |  | | | |  | | | |
| T1 | Tumor limited to the pancreas 2 cm or less in greatest dimension | | | | Tumor limited to the pancreas, 2 cm or less in greatest dimension | | | | Tumor limited to the pancreas, 2 cm or less in greatest dimension | | | |
| T2 | Tumor limited to the pancreas more than 2 cm in greatest dimension | | | | Tumor limited to the pancreas, more than 2 cm in greatest dimension | | | | Tumor limited to the pancreas, more than 2 cm in greatest dimension | | | |
| T3 | Tumor extends directly into any of the following: duodenum, bile duct, peripancreatic tissues | | | | Tumor extends beyond the pancreas but without involvement of the celiac axis or the superior mesenteric artery | | | | Tumor extends beyond the pancreas but without involvement of the celiac axis or the superior mesenteric artery | | | |
| T4 | Tumor extends directly into any of the following: stomach, spleen, colon, adjacent large vessels | | | | Tumor involves the celiac axis or superior mesenteric artery (unresectable primary tumor) | | | | Tumor involves the celiac axis or superior mesenteric artery (unresectable primary tumor) | | | |
| **TNM - N** |  | | | |  | | | |  | | | |
| N0 | No regional lymph node metastasis | | | | No regional lymph node metastasis | | | | No regional lymph node metastasis | | | |
| N1 | N1a: metastasis in a single regional lymph node  N1b: metastasis in multiple regional lymph node | | | | Regional lymph node metastasis | | | | Regional lymph node metastasis | | | |
| **TNM - M** |  | | | |  | | | |  | | | |
| M0 | No distant metastasis | | | | No distant metastasis | | | | No distant metastasis | | | |
| M1 | Distant metastasis | | | | Distant metastasis | | | | Distant metastasis | | | |
| **Extension of disease** | | | | | | | | | | | | |
| **Abbreviated staging** | **Stage** | **T** | **N** | **M** | **Stage** | **T** | **N** | **M** | **Stage** | **T** | **N** | **M** |
| *In situ* | Stage 0 | Tis | N0 | M0 | Stage 0 | Tis | N0 | M0 | Stage 0 | Tis | N0 | M0 |
| Potentially resectable | Stage I | T1  T2 | N0 | M0 | Stage IA | T1 | N0 | M0 | Stage IA | T1 | N0 | M0 |
|  | Stage II | T3 | N0 | M0 | Stage IB | T2 | N0 | M0 | Stage IB | T2 | N0 | M0 |
|  | Stage III | T1  T2  T3 | N1 | M0 | Stage IIA | T3 | N0 | M0 | Stage IIA | T3 | N0 | M0 |
|  |  |  |  |  | Stage IIB | T1  T2  T3 | N1 | M0 | Stage IIB | T1  T2  T3 | N1 | M0 |
| Locally advanced | Stage IVA | T4 | Any | M0 | Stage III | T4 | Any | M0 | Stage III | T4 | Any | M0 |
| Metastatic | Stage IVB | Any | Any | M1 | Stage IV | Any | Any | M1 | Stage IV | Any | Any | M1 |

**Supplementary table 2.** Frequency of surgery for patients with potentially resectable pancreatic cancer and chemotherapy for patients with metastatic pancreatic cancer.

|  |  | **Setting** | | |
| --- | --- | --- | --- | --- |
| **Patients with potentially resectable disease** | **All Patients**  **(N = 1,415)** | **Insurance**  **(N = 139)** | **Public**  **(N = 396)** | **Private**  **(N = 56)** |
| **Surgery (%)^#^**  Yes  No | 1,011 (71.4)  404 (28.6) | 105 (75.5)  34 (24.5) | 266 (67.2)  130 (32.8) | 42 (75.0)  14 (25.0) |
| **Patients with metastatic disease** | **All Patients**  **(N = 3,128)** | **Insurance**  **(N = 226)** | **Public**  **(N = 1,123)** | **Private**  **(N = 102)** |
| **Chemotherapy (%)^@^**  Yes  No | 1,739 (55.6)  1,389 (44.4) | 174 (77.0)  52 (23.0) | 595 (53.0)  528 (47.0) | 43 (42.2)  59 (57.8) |

^#^ P-value for the difference among Insurance, Public, and Private = 0.12 (Fisher exact test).

^@^ P-value for the difference among Insurance, Public, and Private < 0.001 (Fisher exact test).

**Supplementary table 3.** Follow-up according to health care funding setting.

| **Setting** | **N** | **N of events** | **Overall survival**  **Median (95% CI)** – **months** |
| --- | --- | --- | --- |
| **Insurance** | 484 | 347 | 36.1 (33.4 – 41.2) |
| **Public** | 2,258 | 2,116 | 68.5 (61.2 – 78.3) |
| **Private** | 199 | 41 | 4.1 (1.5 – 6.3) |

CI: Confidence interval.

**Supplementary table 4.** Unadjusted overall survival estimates according to patient and tumor characteristics.

|  | **All tumors**  **(N = 6,855)** | | | | |
| --- | --- | --- | --- | --- | --- |
|  | **Median overall survival**  **(months)** | **Overall survival**  **95% CI**  **(months)** | **1-year overall survival rate (%)** | **3-year overall survival rate (%)** | **5-year overall survival rate (%)** |
| **Age (years)**  < 50  50 – 59  60 – 69  ≥ 70 | 7.0  5.9  4.9  3.7 | 6.3 – 7.7  5.3 – 6.5  4.5 – 5.4  3.4 – 3.9 | 34.3  29.9  26.6  20.0 | 12.0  8.8  8.7  5.3 | 8.8  5.0  4.7  2.8 |
| **Gender**  Male  Female | 4.8  5.0 | 4.4 – 5.2  4.7 – 5.3 | 24.9  27.9 | 6.9  9.5 | 3.9  5.7 |
| **Payment source**  Insurance  Public  Private | 11.9  4.4  19.2 | 10.2 – 13.7  4.1 – 4.8  13.2 – NA | 49.7  23.3  66.4 | 18.5  8.0  40.5 | 11.5  5.0  0.0 |
| **Formal education**  Illiterate  Elementary/Middle School  High School  Graduate degree | 3.4  3.9  5.9  10.1 | 2.7 – 3.8  3.7 – 4.1  5.2 – 6.6  9.0 – 11.8 | 16.1  20.8  29.2  44.4 | 6.0  5.9  8.6  17.2 | 3.5  3.3  6.3  10.2 |
| **Extension of disease**  Potentially resectable  Locally advanced  Metastatic | 14.3  7.6  3.7 | 13.0 – 15.8  7.0 – 8.7  3.6 – 3.9 | 54.9  35.8  16.6 | 24.6  8.0  3.0 | 16.1  3.4  1.1 |
| **Anatomic location**  Pancreatic head (C25.0)  Pancreatic body (C25.1)  Pancreatic tail (C25.2)  Pancreatic duct (C25.3)  Langerhans' islets (C25.4)  Other specified parts (C25.7)  Superposed lesion (C25.8)  NOS (C25.9) | 5.3  6.0  3.9  4.8  NA  5.4  4.5  4.4 | 5.0 – 5.8  5.3 – 7.2  3.2 – 5.0  2.1 – 12.0  NA - NA  2.1 – 17.3  3.5 – 6.1  4.1 – 4.8 | 27.6  31.4  25.5  25.0  NA  33.6  28.3  24.0 | 8.3  7.4  10.8  18.8  NA  16.8  8.1  7.4 | 4.2  2.5  6.0  18.8  NA  11.2  6.8  4.8 |
| **Pathological subtype**  Epithelial  Acinar  Ductal or non-specified  carcinoma  Intestinal  Mucinous  Miscellanea  Adenosquamous/SCC  Non-classified  Non-classified | 5.8  10.8  5.7  4.9  10.6  10.2  3.7  2.0 | 5.5 – 6.0  6.2 – 21.5  5.3 – 6.0  3.9 – 5.8  7.9 – 15.7  6.4 – 13.6  2.6 – 5.9  1.7 – 2.2 | 28.9  36.8  27.8  28.9  48.7  46.6  19.7  11.3 | 9.0  16.4  8.2  8.1  21.8  24.0  5.9  3.1 | 5.2  0.0  4.5  3.9  16.2  18.5  3.0  2.1 |

NOS: Not otherwise specified.

SCC: Squamous cell carcinoma.

**Supplementary table 5.** Median and actuarial overall survival according to staging and source of payment.

|  | **Potentially resectable** | | | **Locally advanced** | | | **Metastatic** | | |
| --- | --- | --- | --- | --- | --- | --- | --- | --- | --- |
|  | **Insurance**  **(N = 131)** | **Public**  **(N = 389)** | **Private**  **(N = 50)** | **Insurance**  **(N = 57)** | **Public**  **(N = 305)** | **Private**  **(N = 14)** | **Insurance**  **(N = 221)** | **Public**  **(N = 1113)** | **Private**  **(N = 95)** |
| **Overall Survival - Months**  **Median (95% CI)** | 28.0 (18.1 – 37.8) | 13.3  (11.8 – 16.7) | NR  (14.2 – NR) | 14.5  (12.7 – 19.4) | 7.3  (6.4 – 8.9) | 44.8  (NR – NR) | 7.0  (5.3 – 9.3) | 3.7  (3.2 – 3.9) | 17.1  (7.6 – NR) |
| **1-year Overall Survival Rate – %** | 70.4 | 54.0 | 84.3 | 67.7 | 33.4 | 100 | 34.3 | 14.4 | 52.3 |
| **3-year Overall Survival Rate – %** | 41.6 | 28.6 | - | 16.8 | 8.7 | 100 | 6.7 | 2.9 | - |
| **5-year Overall Survival Rate – %** | 32.1 | 18.2 | - | 6.7 | 5.2 | - | - | 1.5 | - |
| **p value** | < 0.001 | | | < 0.001 | | | < 0.001 | | |

NR: Not Reached.

**Supplementary table 6.** Cox Proportional Hazard regression for overall survival (complete case analysis; N = 1,503 for multivariate analysis).

|  | **Univariate analysis** | | | **Multivariate analysis** | | |
| --- | --- | --- | --- | --- | --- | --- |
| **Variable** | **HR** | **95%CI** | **p** | **HR** | **95%CI** | **p** |
| **Age group (years)^$^**  < 50  50 – 59  60 – 69  ≥ 70 | 1.00  1.14  1.23  1.50 | 1.04 – 1.24  1.13 – 1.34  1.38 – 1.64 | 0.004  < 0.001  < 0.001 | 1.00  0.94  1.10  1.22 | 0.77 – 1.14  0.91 – 1.32  1.00 – 1.48 | 0.510  0.335  0.052 |
| **Gender**  Male  Female | 1.00  0.90 | 0.86 – 0.95 | < 0.001 | 1.00  0.79 | 0.71 – 0.89 | < 0.001 |
| **Period**  2000-2004  2005-2009  2010-2014 | 1.00  1.08  0.93 | 1.00 – 1.16  0.86 – 0.99 | 0.044  0.027 | 1.00  0.85  0.76 | 0.67 – 1.10  0.61 – 0.94 | 0.214  0.012 |
| **Formal education**  Illiterate  Elementary/Middle  School  High School  Graduate degree | 1.00  0.89  0.69  0.59 | 0.79 – 0.99  0.61 – 0.78  0.43 – 0.56 | 0.028  < 0.001  < 0.001 | 1.00  1.03  0.88  0.74 | 0.81 – 1.32  0.67 – 1.16  0.55 – 1.00 | 0.809  0.371  0.048 |
| **Source of payment**  Insurance  Public  Private | 1.00  1.78  0.61 | 1.59 – 1.99  0.44 – 0.85 | < 0.001  0.003 | 1.00  1.42  0.33 | 1.16 – 1.73  0.20 – 0.54 | 0.001  < 0.001 |
| **Extension of disease**  Potentially  resectable  Locally advanced  Metastatic | 1.00  1.57  2.78 | 1.52 – 1.83  2.59 – 2.99 | < 0.001  < 0.001 | 1.00  1.57  2.82 | 1.30 – 1.88  2.42 – 3.27 | < 0.001  < 0.001 |
| **Treatment**  No  Yes | 1.00  0.33 | 0.31 – 0.34 | < 0.001 | 1.00  0.31 | 0.27 – 0.36 | < 0.001 |

^$^Proportionality of hazards violated for the variable Age group and for the global test (p = 0.05).

# **Supplementary figures**

**Supplementary figure 1.** Study population.


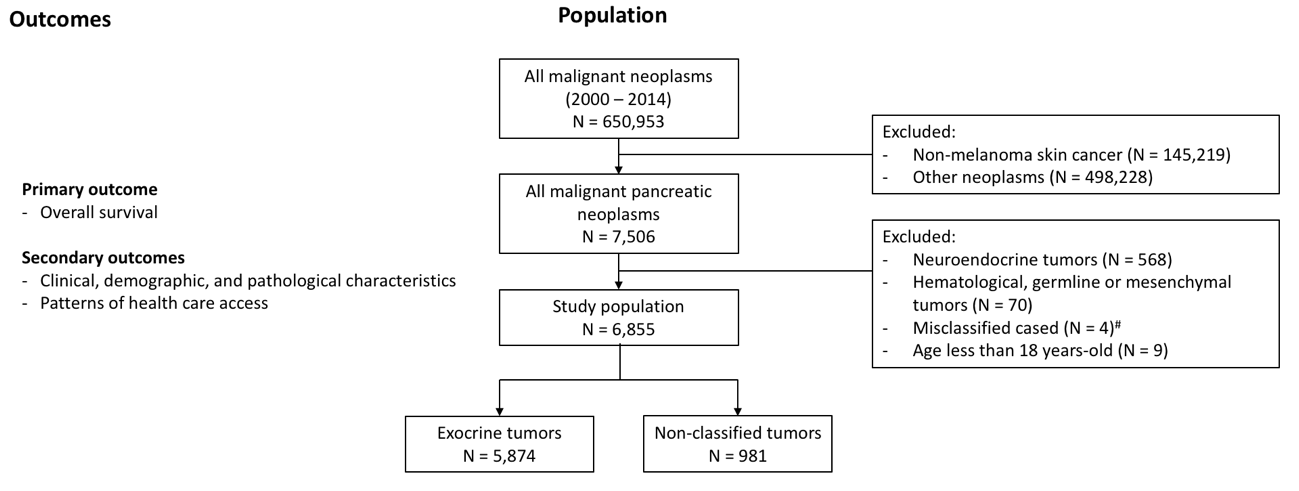


^#^ Misclassified cases included two patients with transitional cell carcinomas and two with adenocarcinomas arising from polyps.

# **References**

1. White, I.R. & P. Royston. *Imputing missing covariate values for the Cox model.* *Stat. Med.* **28**(15), 1982-98 (2009).

2. Cleves, M., Gould, W.W. & Marchenko, Y.V. *The Cox proportional hazard model: Cox model with missing data: multiple imputation* in *An Introduction to Survival Analysis Using Stata* 172-78 (Stata Press, 2016).

3. No author listed. *Stata multiple-imputation* in *Stata 13 Base Reference Manual* 1-373 (Stata Press, 2013).

4. Fleming I.D., Henson D.E., Hutter R.V.P., Kennedy B.J., Murphy G.P., O’Sullivan B, Sobin LG, Yarbro JW (Eds.). *Exocrine Pancreas* in *AJCC Cancer Staging Manual* 121-26 (1997).

5. Greene FL, P.D., Fleming ID, Fritz AG, Balch CM, Haller DG Morrow M. *Exocrine Pancreas* in *AJCC Cancer Staging Manual*. 157-64 (Springer, 2002).

6. Edge S.B., Compton C.C., Fritz A.G., Greene F.L., Trotti III, A. (Eds.). *Exocrine and Endocrine Pancreas* in *AJCC Cancer Staging Manual*. 241-49 (Springer, 2010).
